# Supplementary material for: Improved transcriptome assembly using a hybrid of long and short reads with StringTie
Source: PLoS Comput Biol. 2022 Jun 1;18(6):e1009730. doi: 10.1371/journal.pcbi.1009730 (PMC9191730; doi:10.1371/journal.pcbi.1009730)
Supplement: S1 Table — Error rates of all long-read datasets before (a) and after correction (b) with TALC. (DOCX) [file pcbi.1009730.s004.docx]

**Table S1a. Error rates of all long-read datasets before correction with TALC.**

| **Sample** | **Species** | **Sequencing Type** | **Indel Rate (%)** | **Mismatch**  **Rate**  **(%)** | **Total Error Rate**  **(%)** |
| --- | --- | --- | --- | --- | --- |
| ERR2680375 | *M. musculus* | ONT dRNA | 12.3 | 4.9 | 17.2 |
| ERR2680377 | *M. musculus* | ONT cDNA | 9.9 | 4.4 | 14.3 |
| ERR2680379 | *M. musculus* | ONT cDNA | 11.3 | 4.6 | 15.9 |
| ERR3764345 | *A. thaliana* | ONT dRNA | 11.1 | 4.6 | 15.7 |
| ERR3764349 | *A. thaliana* | ONT dRNA | 11.3 | 5.0 | 16.3 |
| ERR3764351 | *A. thaliana* | ONT dRNA | 11.2 | 5.0 | 16.2 |
| NA12878-cDNA | Human | ONT cDNA | 11.2 | 4.4 | 15.6 |
| NA12878-DirectRNA | Human | ONT dRNA | 8.2 | 2.7 | 10.9 |
| SRR1163655 | Human | PacBio cDNA | 2.8 | 0.4 | 3.2 |
| Simulated-dRNA | Human | Simulated ONT dRNA | 7.8 | 2.6 | 10.4 |

**Table S1b**. **Error rates of all long-read datasets after correction with TALC.**

| **Sample** | **Species** | **Sequencing Type** | **Indel Rate (%)** | **Mismatch**  **Rate**  **(%)** | **Total Error Rate**  **(%)** |
| --- | --- | --- | --- | --- | --- |
| ERR2680375 | *M. musculus* | ONT dRNA | 3.3 | 1.4 | 4.7 |
| ERR2680377 | *M. musculus* | ONT cDNA | 3.5 | 1.3 | 4.8 |
| ERR2680379 | *M. musculus* | ONT cDNA | 3.0 | 1.7 | 4.7 |
| ERR3764345 | *A. thaliana* | ONT dRNA | 3.5 | 1.5 | 5.0 |
| ERR3764349 | *A. thaliana* | ONT dRNA | 2.9 | 1.3 | 4.2 |
| ERR3764351 | *A. thaliana* | ONT dRNA | 2.8 | 1.3 | 4.1 |
| NA12878-cDNA | Human | ONT cDNA | 4.4 | 1.9 | 6.3 |
| NA12878-DirectRNA | Human | ONT dRNA | 2.2 | 0.9 | 3.1 |
| SRR1163655 | Human | PacBio cDNA | 1.4 | 0.4 | 1.8 |
