# Supplementary material for: Improved transcriptome assembly using a hybrid of long and short reads with StringTie
Source: PLoS Comput Biol. 2022 Jun 1;18(6):e1009730. doi: 10.1371/journal.pcbi.1009730 (PMC9191730; doi:10.1371/journal.pcbi.1009730)
Supplement: S2 Table — (DOCX) [file pcbi.1009730.s005.docx]

| Table S2. Percentage of reads that are full-length isoforms and the number of unique full-length isoforms captured in each long-read dataset. We define a full-length isoform as a read that spans all exon/intron boundaries of a multi-exon transcript or a read that spans at least 80% of a single-exon transcript. | | | | |
| --- | --- | --- | --- | --- |
| **Sample** | **Species** | **Sequencing Type** | **% Full-length Isoforms** | **Number of Unique Full-length Isoforms** |
| ERR2680375 | *M. musculus* | ONT dRNA | 25.4 | 22882 |
| ERR2680377 | *M. musculus* | ONT cDNA | 36.2 | 34441 |
| ERR2680379 | *M. musculus* | ONT cDNA | 40.0 | 14824 |
| ERR3764345 | *A. thaliana* | ONT dRNA | 60.6 | 31479 |
| ERR3764349 | *A. thaliana* | ONT dRNA | 66.5 | 33132 |
| ERR3764351 | *A. thaliana* | ONT dRNA | 67.2 | 29618 |
| NA12878-cDNA | Human | ONT cDNA | 51.1 | 40368 |
| NA12878-DirectRNA | Human | ONT dRNA | 47.3 | 57563 |
| SRR1163655 | Human | PacBio cDNA | 42.8 | 67900 |
| Simulated-dRNA | Human | Simulated ONT dRNA | 35.1 | 32324 |
