# Supplementary material for: Improved transcriptome assembly using a hybrid of long and short reads with StringTie
Source: PLoS Comput Biol. 2022 Jun 1;18(6):e1009730. doi: 10.1371/journal.pcbi.1009730 (PMC9191730; doi:10.1371/journal.pcbi.1009730)
Supplement: S1 File — (DOCX) [file pcbi.1009730.s006.docx]

**Supplementary Information File S1**

**Equal Coverage Simulation Results**

To ensure that the improvements in the hybrid-read assemblies were not simply due to increased coverage, we simulated more long and short reads such that the coverage of the long, short, and hybrid-read datasets are approximately equal (see Methods). We then computed the precision and sensitivity of the original simulated dataset (Supplementary Figure 1A) and this equal coverage dataset (Supplementary Figure 1B) using the full set of expressed transcripts as the reference. In this case, we did not require a transcript to be fully covered by the simulated reads in order to count it as truly expressed since the set of fully covered transcripts is different in each dataset. In the original dataset where the hybrid-read coverage is the combination of the long and short read coverage, the average increase in precision of the mixed-read assemblies over the short-read assemblies was 16.3% and the average increase in sensitivity was 21.6%. The average increase in precision and sensitivity over the long-read assemblies was 13.1% and 25.8% respectively. In the equal coverage dataset, the average increase in precision of the mixed-read assemblies over the short-read assemblies was 23.6% and the increase in sensitivity was 20.9% The average increase in precision and sensitivity of the mixed-read assemblies over the long-read assemblies was 13.4% and 20.5%. This shows that the improvements from hybrid-read assembly are not simply explained by the increase in read coverage.

**
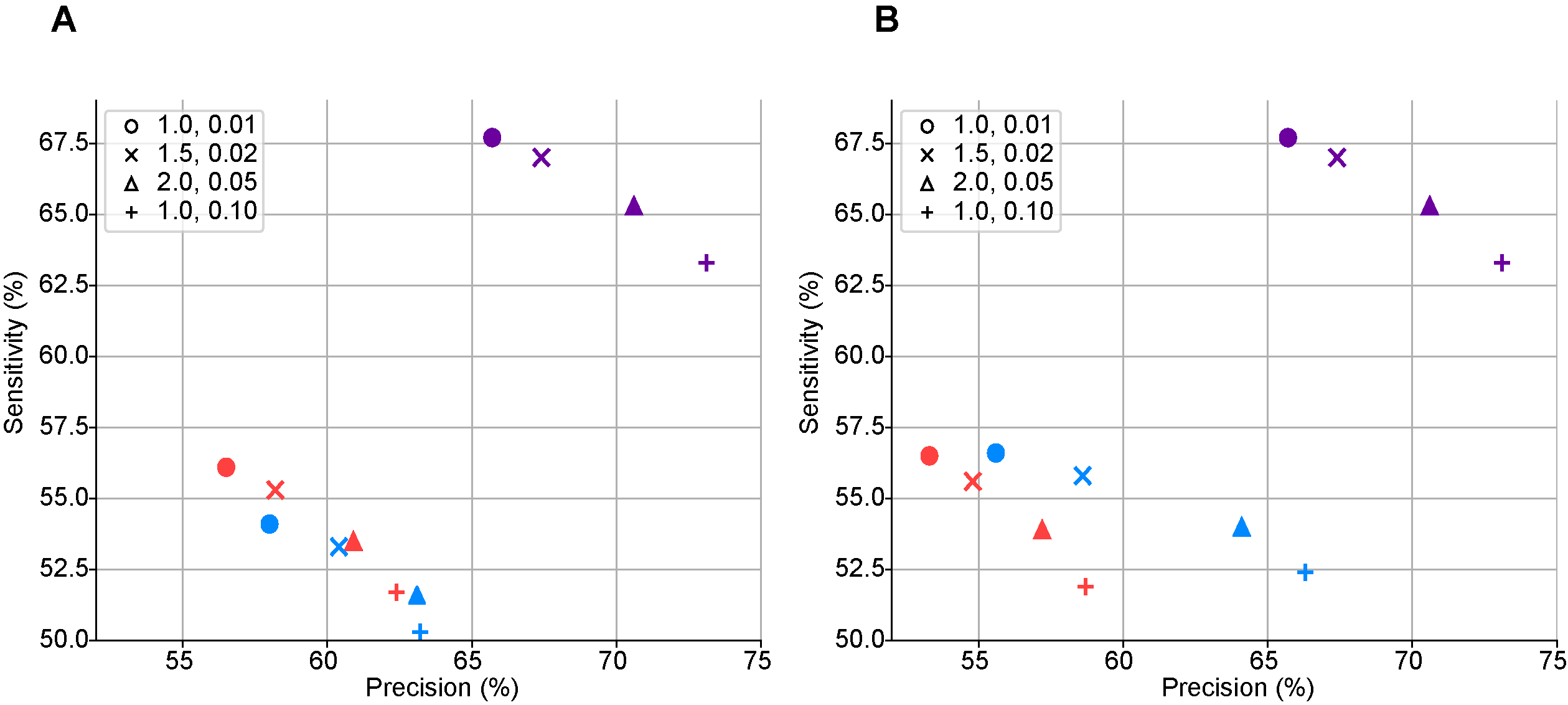
**


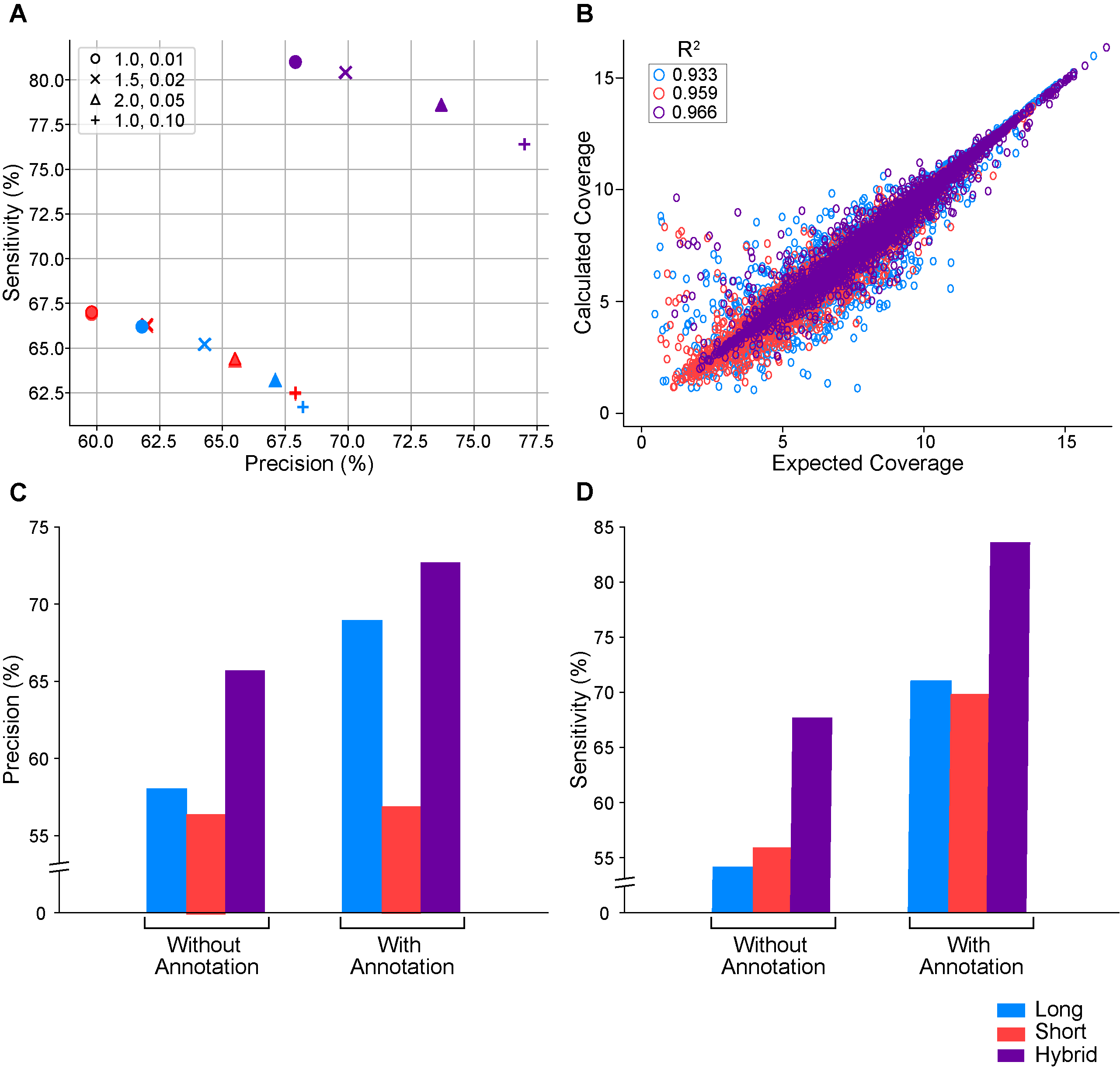


Figure S1. Transcript assembly accuracy at all expressed loci in short, long, and hybrid simulated data sets. A) Sensitivity and precision of the assemblies created from the original dataset where the hybrid read coverage is the combination of the long read and the short read coverage. B) Sensitivity and precision of the assemblies created from the dataset where the coverage of the short, long, and hybrid reads is approximately equal. The two StringTie parameters varied were the minimum read coverage allowed for a transcript (-c) and the minimum isoform abundance as a fraction of the most abundant transcript at a given locus (-f). Each shape represents a different combination of -c,-f parameters with the values indicated in the legend.
