# Supplementary material for: Improved transcriptome assembly using a hybrid of long and short reads with StringTie
Source: PLoS Comput Biol. 2022 Jun 1;18(6):e1009730. doi: 10.1371/journal.pcbi.1009730 (PMC9191730; doi:10.1371/journal.pcbi.1009730)
Supplement: S2 File — (DOCX) [file pcbi.1009730.s007.docx]

**Supplementary Information File S2**

**Hybrid transcriptome assembly of short and long read data from the HepG2 cell line**

We analyzed sample-matched long and short reads from the HepG2 cell line which is a human liver cancer cell line. Short reads from 3 replicates (SRR16071312, SRR16071313, SRR16071314) were generated with an Illumina NovaSeq and long reads from 5 replicates (SRR16071311, SRR16071315, SRR16071316, SRR16071317, SRR16071318) were generated with direct RNA ONT sequencing using SQK-RNA002 chemistry and basecalled with Guppy version 5.0.7. For both the long reads and the short reads, we aligned the reads from all replicates and merged the resulting alignments together using samtools merge. We then created long, short, and hybrid-read assemblies and compared the accuracy as previously described in the main text . Results are shown in Supplementary Figure 3A. We saw that compared to the long-read assembly the hybrid-read assembly had a 9.0% increase in precision and a 26.4% increase in the number of annotated transcripts assembled. Compared to the short-read assembly, the hybrid-read assembly had an increase in precision of 6.7% and an increase in the number of annotated transcripts assembled of 30.3%. We also examined the level of support in the RefSeq annotation of the assembled transcripts in each assembly and saw that the hybrid-read assembly contains the most RefSeq curated (well supported) transcripts and RefSeq predicted (poorly supported) transcripts (Supplementary Figure 3B).

**
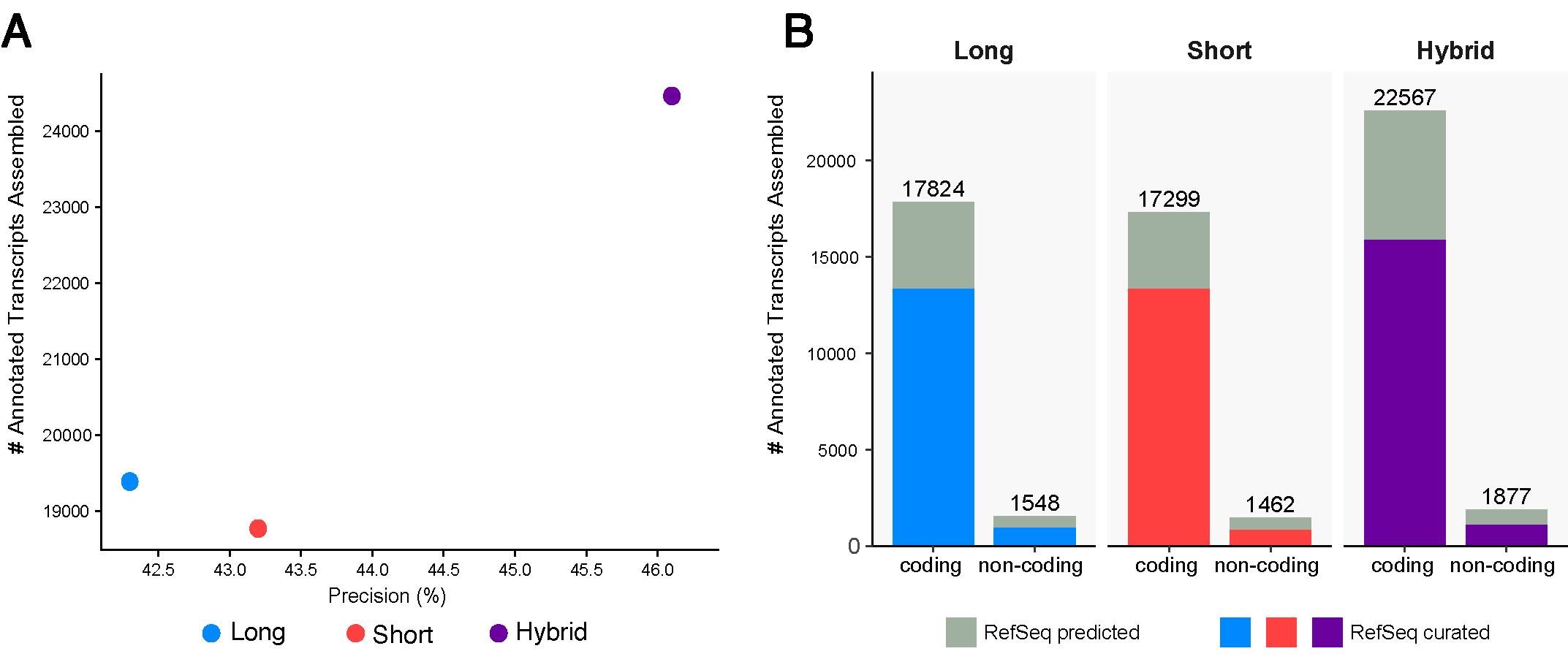
**

**Figure S3. Transcript assembly accuracy on RNA-seq data from the HepG2 cell line.**  **A)** Precision and number of annotated transcripts assembled from long, short, and hybrid-read assemblies generated from reads from the HepG2 cell line. **B)** The number of predicted and curated transcripts assembled in the long, short, and hybrid-read assemblies generated from reads from the HepG2 cell line. Predicted means that the transcript is poorly supported according to the RefSeq annotation and curated means the transcript is highly supported.
