# Supplementary material for: Improved transcriptome assembly using a hybrid of long and short reads with StringTie
Source: PLoS Comput Biol. 2022 Jun 1;18(6):e1009730. doi: 10.1371/journal.pcbi.1009730 (PMC9191730; doi:10.1371/journal.pcbi.1009730)
Supplement: Attachment — Submitted filename: hybrid_read_stringtie_review_responses.docx [file pcbi.1009730.s008.docx]

**Answers to the reviewers**

We thank the reviewers for taking the time to review our manuscript. We believe that their suggestions have indeed strengthened the manuscript. See our responses to each of their points below.

**Reviewer 1**

**General comments:**

Shumate et al describe a new release of the transcriptome assembler StringTie. In this manuscript, they set out to determine whether hybrid assemblies generated with Illumina and Nanopore data result in greater accuracy than assemblies generated with just one data type. In principle, the results for three higher eukaryotes support their claims although I think a deeper analysis of the data would prove more informative and strengthen the manuscript considerably.

**Major comments:**

**1. Abstract**

The Abstract describes long read sequencing as low throughput. While this is true when comparing a MinION to a high output Illumina platform (NextSeq, HiSeq, NovaSeq) but now when comparing against the GridION or PromethION

We have removed the statement describing long-read sequencing as low-throughput (line 17).

**2. Introduction**

a. The first is that proteomic diversity and phenotypic complexity is not exclusively limited to higher eukaryotes. Indeed many plants, bacteria, and viruses exhibit remarkably complex transcriptomes.

We have removed the part about proteomic diversity and phenotypic complexity and now simply state that transcriptome assembly is more challenging in eukaryotes due to alternative splicing (lines 50-52).

b. The second is that the error-rate quoted for nanopore direct RNA-Seq is way out of date. Indeed the most recent chemistries (SQK-RNA002) combined with later Guppy versions (v4 onwards) reduce the error rate to ~3-4%.

We were unable to find any published data regarding the error rates of the most recent chemistry and latest Guppy version, although we have heard from Oxford Nanopore reps that they can achieve much lower error rates. After much searching, we found one publicly available dataset using SQK-RNA002 chemistry basecalled with Guppy v5; however (to our surprise), the error rate was still >10% (see more details in response to comment 3b). To avoid quoting an incorrect number, we changed the statement to say that “long reads have a high error rate relative to illumina short reads” (line 71).

c. Finally, the authors state that practical limitations impede the ability to capture full-length transcripts. It would help significantly if the authors could put actual numbers on this. For instance, in our hands (and others), we typically observe between 30-60% full-length transcripts (SQK-RNA002 + Guppy v4).

Our observations match the reviewer’s, in the sense that we observe between 25-67% full-length isoform coverages by the long reads. We added this information for all long-read datasets in Supplementary Table S2 (referenced in lines 214-216).

**3. Results**

a. I have many questions about the underlying data, both simulated and real. In the case of the ONT data, it is imperative to include information on the Chemistry (SQK-RNA001 or SQK-RNA001 for DRS), basecalling (which Guppy versions) as this will very clearly impact on the accuracy of the final ONT fastq files, and the resulting alignments. Indeed one suspect for the latest chemistry + basecaller combinations that the numbers of artefacts in DRS datasets (false exons, incorrect splice junctions) will significantly reduce. These queries also extend to the simulated data. What do the simulated DRS datasets look like? Are they modelled on SQK-RNA001 or SQK-RNA002 chemistry? Similar questions pertain to the Illumina data. For instance, was this all poly(A) selected or were these ribo-depleted libraries?

We have added chemistry and basecaller information for each dataset in Table 1. All Illumina libraries were poly(A) selected and we have added this statement to the text. Lastly, we have stated in the methods section that the NA12878-dRNA reads were used to build a model for the simulated data. As shown in the updated Table 1, the NA12878-dRNA reads were generated with SQK-RNA001 chemistry (lines 279-282).

b. Extending this further, do the authors see any difference in transcriptome assembly quality when comparing chemistry or basecaller versions? While it is abundantly clear that hybrid assembly is still the best option, it would be valuable to know whether further improvements to chemistry/basecalling would increase the relative accuracy of DRS alone approaches.

After much searching we were only able to locate one dataset with short reads and long reads from the same sample where the long reads were generated with SQK-RNA002 chemistry and basecalled with Guppy v4 or higher. This dataset comes from the HepG2 cell line and is described in more detail in the supplement. Accuracy of long, short, and hybrid-read assemblies created from this dataset are shown in Supplementary Figure S3A. Compared to the NA12878-dRNA (generated with SQK-RNA001) results, the relative increase in precision of the mixed reads over the long reads dropped slightly from 12.1% to 9.0%. The relative increase in the number of annotated transcripts assembled also dropped from 26.4% to 23.2%. While the relative accuracy does drop with the newer chemistry/basecaller versions, the hybrid-read assembly is still clearly better.

Important to note is that the error rate of this new dataset is higher than expected based on the reviewer’s observation that SQK-RNA002 chemistry and Guppy v4 achieves an error rate of 3-4%. We calculated the error rate of this dataset to be 10.6% which is only slightly below the NA12878-dRNA reads which have an error rate of 10.8%. We believe that a stronger case for the benefits of hybrid-read assembly, even as error rates of long reads inevitably decline, is shown with the accuracy of the TALC-corrected long-read assemblies compared to the hybrid-read assemblies. TALC correction reduced the error rate to as low as 1.8% (in the human PacBio reads) and yet we still observe higher accuracy in the hybrid-read assembly (Figure 3I).

We added a discussion of the results on the long data sets generated with the newer chemistry to the main manuscript too (lines 326-334) as well as to the Supplementary File S2.

c. For the real world data, it would great if table 1 were to include more information on the source/cell types sequenced and also how the various datasets link together. For instance, Fig 3 pairs together Nanopore and Illumina datasets but it is now clear how/why these pairs specifically were chosen.

We have added a sentence clarifying that the long and short reads in each pair are from the same sample. We also now state directly that the human reads all come from the NA12878 cell line and the mouse reads are from brain and liver tissue (lines 206-209). The tissue type for each mouse sample has been added to Table 1 (lines 279-282).

d. A deeper dive into the assembled transcriptomes is warranted. For instance, transcripts in the existing HG38 annotation are variously classified according to support (experimental, theoretical, etc). It would be interesting to know, for a given hybrid assembly, how many poorly supported transcripts in the existing annotation are actually confirmed by hybrid assembly?

We have now included this analysis for the HepG2 assemblies in Supplementary Figure S3B.

e. The comparison to TALC is useful but what about other assemblers that offer ‘hybrid-like’ assembly? For instance, FLAIR uses Illumina data to infer splice junctions that are then ‘corrected’ in nanopore-derived alignments. While I suspect the StringTie approach to be faster and more accurate, it would be useful to demonstrate this definitively.

We recognize that a comparison to FLAIR is useful; however, we have already demonstrated in a previous StringTie paper (see Kovaka S et al. Genome Biology. 2019) that long-read assembly with StringTie is superior to FLAIR, both in terms of accuracy as well as speed. Given that we show here hybrid-read assembly is superior to long-read assembly with StringTie, we do not feel it is necessary to repeat the FLAIR comparison here.

**4. Methods**

a. Linking back to comment 3a, more information on how NanoSim was run and what the resulting data look like is needed. What % of NanoSim reads are full length and how does this compare to the real-world datasets? What chemistry (SQK-RNA001 or SQK-RNA002) and basecalling (Guppy version) is simulated?

The simulated reads were modeled off of the read-word data (NA12878-dRNA) and the chemistry and basecalling information for this dataset has been added to Table 1. The % of full length reads for the simulated data and the read data are now included in Supplementary Table S2.

**Minor comments:**

1. Line numbers and page numbers would have made this manuscript easier to review! I do however appreciate the inclusion of figures within the manuscript body rather than at the end as this makes it much easier to read.

Apologies for the lack of line and page numbers. We have included these in the re-submission.

2. While I understand the choice of red, blue, purple for the figures, these colours are not easy to differentiate. The authors could consider lightening the shade of each or finding an alternative combination that segregates better.

We have lightened the blue in each of the figures to hopefully differentiate it better from the purple.

3. It would be useful to have the test datasets available (or at least linked to) within the repository. Alternatively, a collection of ‘light’ test datasets containing reads for a just a few select transcripts would be useful for those who which to explore the various parameters and outputs.

We agree that this is a good suggestion. The StringTie package available from github (https://github.com/gpertea/stringtie/) already includes test data sets for all types of input RNA-Seq data (short, long, or both). The users can verify their results on those data sets in order to compare them to what is expected to be assembled at a given locus. In addition, we also provide now all the alignments for the data we used in the manuscript as well as the reads for the simulated data from [ftp://ftp.ccb.jhu.edu//pub/data/stringtie_hybrid_read/](ftp://ftp.ccb.jhu.edu/pub/data/stringtie_hybrid_read/)

4. In the Methods section, it would be useful to spell out the commands used for HiSat2 and MiniMap.

We have edited the methods to include the full commands for both HiSat2 and Minimap2 (lines 559-565).

5. Was any filtering performed on the RefSeq annotations?

No filtering was performed on the RefSeq annotations. The new Supplementary Figure S3B shows that the hybrid assembly identifies more transcripts matching curated annotations in RefSeq.

**Reviewer 2**

**General comments:**

The manuscript " Improved Transcriptome Assembly Using a Hybrid of Long and Short Reads with StringTie" by Schumate et al. describes the authors' latest developments on the StringTie software, incorporating the capability to assemble transcript alignments from both short (tens to hundred base length Illumina reads) and long (several kilobase length PacBio or ONT) RNA-seq reads. The initial version of StringTie (2015, Nature Biotechnology) assembled short RNA-seq read alignments, StringTie2 (2019, Genome Biology) had specializations for assembling long read alignments, and now the latest version of StringTie (perhaps more aptly named StringTie3?) is presented here for co-assembly of short and long read alignments. The authors demonstrate with both simulated and real RNA-seq data that this latest StringTie provides more accurate assemblies when both the short and long read alignments are available.

StringTie continues to be one of the most popular genome-guided alignment assemblers for reconstructing transcripts, and is my personal favorite for routine use in my own work. I'm pleased to see that this new functionality of assembling both short and long reads is now integrated.

The manuscript is very well written and the experiments performed are all relevant and necessary for this work. I do have a few critical comments and suggestions as follows:

We thank the reviewer for his or her positive comments.

**Major comments:**

1. When combining short read alignments with the long read alignments, the effective coverage of transcripts will increase. Within low to moderately expressed transcripts, increased effective coverage would be expected to yield increased rates of full reconstruction, separately from read types provided. The authors might provide some supplementary study to demonstrate that the improved accuracy for reconstruction with hybrid data is more due to having the hybrid data types available rather than due to increased effective alignment coverage. This could be done with simulated data by controlling for total effective coverage when assembling in short-only, long-only, or hybrid data.

Thank you to the reviewer for this helpful suggestion. We have included an experiment where the coverage of the short, long, and hybrid reads is approximately equal. The accuracy results are shown in Supplementary Figure S1 and also discussed in the Results section (lines 172-177). The exact details of the simulation have been added to the methods (lines 542-556) and Supplementary File S1.

2. Figure 1 would ideally show a more impactful example of where both read types are needed to assemble the full-length isoform. In the example shown, a splicing graph based on short read alignments alone looks as though it would contain the full-length splicing pattern, and if it was not assembled by StringTie, it might be due to StringTie parameter settings or StringTie-specific logic. A different, even naive splice graph assembler has the potential to reconstruct the full-length splicing pattern based on just those short reads alone.

We have chosen a different example for Figure 1 where it is hopefully more clear how both read types are needed to correctly assemble the full length isoform (lines 103-138).

3. It is useful that StringTie can make use of 'dirty' long reads (those with high error rates), but it is worth noting that the modern PacBio IsoSeq and ONT transcriptome sequencing methods are currently producing much higher fidelity data. The authors might comment on this in the manuscript. Characteristics of the real long read data being used with StringTie in this manuscript should be made available, perhaps as supplementary materials, to shed insight into the overall quality of those data - ie. are they early error-prone or later higher fidelity long reads being leveraged. In particular, it would be helpful to know characteristics (ie. percent identity or error rates) before and after applying TALC for error correction.

In Figures 2 and 3 that provide accuracy statistics for the different StringTie invocations, it would be helpful to know for the long reads what the full-length sensitivity and specificity would be without doing any StringTie assembly (for example, running a method like cd-hit to reduce redundancy, and examine accuracy statistics for the cd-hit compiled reference isoforms aligned with minimap2). This would provide a nice reference point for defining the baseline quantity of full-length isoforms from which StringTie would further augment through assembly.

We agree that this information is very useful and we have added it to the supplement. Supplementary Table S1 shows the error rates of the long reads before and after correction with TALC. Supplementary Table S2 shows the percentage of reads that are full length isoforms and the number of unique isoforms assembled in each dataset. The details of this analysis have been included in methods in the “Error-rate Calculations and Full-length Isoform Analysis” section (lines 634-649).

Regarding the improving data quality, TALC correction reduced the error rate to as low as 1.8% (in the human PacBio reads) and yet we still observe higher accuracy in the hybrid-read assembly (Figure 3 I). This suggests that even as error rates inevitably decline, hybrid-read assembly will still be beneficial for the foreseeable future. We have added a brief statement about this in the discussion (lines 404-408).

4. The experiments performed with real data involved gathering transcriptome data from public databases. Unlike the simulated data, where the long and short reads appear to be derived from matched data (same genes being expressed), it isn't clear that the real data are similarly matched (so potentially many different genes being expressed between long and short read data). Table 1 doesn't indicate the tissue type that the data correspond to, and I couldn't easily ascertain it from the original source either. Accuracy analysis leveraging these real data were restricted to those genes having long read coverage, although the authors state (indicating data not shown) that results are similar when all loci are examined. There are a couple of issues to comment on here.

First, I encourage the authors to provide (in supplement) results observed when considering all loci. This will address the issue of how much more comprehensive and accurate an assembly derived from StringTie when provided all data as opposed to just short or long reads alone, which would be useful to know in practice.

The data for all loci have been provided in Supplementary Figure S2 (referenced in lines 230-231).

Second, by restricting to only loci with long reads, because the derived samples may be unmatched, there is little assurance that relevant genes expressed with long reads will be expressed in the samples from which the short reads were derived. This disparity might account for lower sensitivity of short-only as compared to long-only in Figures 3G,H. A paucity of short reads for a long-read-covered locus would also presumably impact the ability of TALC for error correction at corresponding loci, but I suspect that has negligible effect on StringTie reconstruction given TALC-related results from other experiments presented here.

All of the pairs of long and short reads are from the same sample and presumably have the same genes expressed. We have added a sentence to the manuscript to clarify this (lines 208-209).

5. Figures 2C,D and Figure 4 involve barplots showing sensitivity and specificity separately instead of plotting together in scatter form (as in Figure 3). I think it's more transparent to assess sensitivity and specificity together in scatter form rather than separately in the barplots, unless there's some specific reason to show them separately here.

We chose to present the data in bar charts because we believe that it is cleaner visually. Combining the data from multiple samples onto one scatter plot becomes hard to read. Alternatively, creating a separate scatter plot for each results in very sparse plots (only 3 points per plot) and a lot of wasted space. We included precision and sensitivity next to each other in each figure to maintain as much transparency as possible while making the data easy to read.

**Minor comments**

1. Abstract: "unable to span multiple exons" -> "rarely span multiple exons" (as 'unable' is not true, and 'rarely' is used elsewhere in the manuscript).

We have edited the abstract to say ‘rarely’ (line 15).

2.Intro: " Additionally, long reads have a high error rate between 13% and 15% [4], and the throughput of long-read RNAseq is much lower than that of short-read RNA-seq." This was a true statement for the earlier state of the technology, but due to recent rapid advances, this is no longer true. This recent advance would be worthy of commenting on in the Intro.

We have removed this statement from the introduction. See response 2b to Reviewer 1 for more information.

3. Reference annotation-assisted assembly has been used by StringTie and earlier methods to leverage reference isoform structures as part of generating a more complete assembly. I encourage the authors to include more information in the methods on how StringTie does this, or reference earlier StringTie paper(s) that describe it sufficiently. In particular, describing how it contributes to improving specificity of reconstruction would be most helpful, as I find it less intuitive than improving sensitivity.

We have included details on how StringTie uses the reference annotation to assemble transcripts (lines 425-433).

4. Please review figure 5 to ensure that the "Noisy" splice graph accurately reflects the read alignments. I think the bottom pink arrow needs to be shifted to the right.

We thank the reviewer for noticing this. We have corrected the splice graph in Figure 5.

5. Please be sure to make inputs (aligned bams, simulated fastqs, etc.) available on a data sharing site.

We provide all alignments for the data we used in the manuscript as well as the reads for the simulated data (in fastq format) from [ftp://ftp.ccb.jhu.edu//pub/data/stringtie_hybrid_read/](ftp://ftp.ccb.jhu.edu/pub/data/stringtie_hybrid_read/) (mentioned on lines 654-655).
