# Supplementary material for: Improved transcriptome assembly using a hybrid of long and short reads with StringTie
Source: PLoS Comput Biol. 2022 Jun 1;18(6):e1009730. doi: 10.1371/journal.pcbi.1009730 (PMC9191730; doi:10.1371/journal.pcbi.1009730)
Supplement: Attachment — Submitted filename: StringTie_response_to_reviewers_April29.docx [file pcbi.1009730.s009.docx]

Reviewer #1: I am quite satisfied with the detailed and considerate responses provided by authors. I thus recommend this paper be accepted for publication.

Reviewer #2: I appreciate the authors responses to reviewers comments and the manuscript is substantially improved and strengthened as a result of the additional experiments performed and related revisions. I have only a few more technical comments that the authors may further consider.

Thank you to both of the reviewers for taking the time to review our manuscript and provide valuable feedback. Below are our responses to specific comments.

The results provided in Figure 4 indicate the accuracy differences between methods while leveraging the reference annotation as a guide. It would be useful to see the difference in accuracy for each sample as a result of including vs. excluding the reference annotations as well, as shown for simulated data in Figure 2C,D, providing those results in the supplementary materials if they are not easily integrated into the main figure.

We have updated Figure 4 to include the accuracy results of the assemblies both with and without guide-annotation for each sample.

In Figure 2C, it is unusual or unexpected that the precision appears largely unchanged for short reads when annotations are added as a guide. This would be worth reviewing and understanding further.

In our experience this is not an unexpected result with short read data. Since short reads do not span full length transcript structures, annotated isoforms can appear as covered by reads even though they are not expressed (i.e. when the beginning of a transcript is mistakenly assembled with the end of another transcript). Indeed, upon reviewing our assemblies, we found that using the annotation as a guide results in more annotated transcripts being assembled even though they are not actually expressed, which increases the number of false positives. However, the precision does not decrease and pretty much stays the same due to the fact that more actually expressed annotated transcripts are assembled as well. This does not appear to be a problem with the hybrid-read data (or the long-read data) which further supports our main conclusion that hybrid-read data is more accurate with and without annotation. A brief explanation of this result was added on lines 173-177.

The results statement "The use of hybrid-read data plus annotation had an increase in precision of 10.7% and an increase in sensitivity of 23.5% as compared to using short reads plus annotation, which in turn was superior to using long reads plus annotation." on page 9 is not clear from Fig 2 with respect to how the short and long read (non-hybrid executions) rank compared to each other.

Thank you to the reviewer for catching this and bringing it to our attention. This is indeed an error. The 10.7% increase in precision and 23.5% increase in sensitivity is compared to hybrid-read data without annotation rather than the short-read plus annotation. We have updated the statement to say “The use of hybrid-read data plus annotation had an increase in precision of 10.7% and an increase in sensitivity of 23.5% as compared to the hybrid-read data assembled without annotation.” (lines 178-179).

In Figure 3 and particularly in the new Supplementary Figure 2, the heavily increased precision for corrected long reads is strikingly different for Arabidopsis as compared to human and mouse. Similarly, the lower precision for hybrid model compared to corrected long reads is surprising and deserves explanation if possible. One feature of the Arabidopsis genome compared to mouse and human would be the compact gene structures and prevalence of retained introns in transcriptome sequencing data. Could this be partly responsible for the different behavior of Stringtie on Arabidopsis? Other reasons?

Due to the different scales on the X-axis for each species, the increased precision of the corrected long reads for Arabidopsis is not as large as it appears visually. In Supplementary Figure 2 (results at all loci), TALC corrected long reads increased the precision of assembly in Arabidopsis by an average of 5.9%, mouse by 4.0% and human by 0.7%. This correlates with the average error rates (16.0% for Arabidopsis, 15.8% for mouse, and 9.8% for human). This indicates that long read correction with TALC leads to larger precision improvements when the error rate is higher, which is the expected result.

Regarding the lower precision for the hybrid-data compared to the corrected long reads, there may be many factors causing this such as the quality of the RNA-seq data, the compact gene structures, the quality of the reference annotations, etc. Any explanation we attempt to provide would only be speculation. The number of annotated transcripts is still much higher with the hybrid-read data and StringTie is much faster than TALC. Because of this we do not believe this changes the overall conclusion of our manuscript which is that hybrid-read assembly with Stringtie is the superior approach when considering precision, the number of annotated transcripts assembled, and the runtime of long-read correction.

I appreciate that the authors added the read error characteristics in Table S1. This is very useful. However, this is limited to mismatches and doesn't include statistics on indels - which are most relevant to the long read data. Please include the indel rates as well, along with methods info on how this was done according to current best practices.

The read error characteristics in Table S1 were the total error rate including both mismatches and indels. We apologize for not making this clear. We have updated the table to include the indel rate, the mismatch rate, and the total error rate and added methods information in lines 608-615. We also separated the pre-correction error rates and the post-correction error rates into two tables (Table S1a and Table S1b) to improve readability.

Minor

Figure 2B: should indicate in the legend that coverage refers to log2(1+coverage) as described in the Methods.

We have added this to the figure caption.

A small suggestion for wording: Page 9 "Although we cannot know exactly which transcript 'molecules' are present in the samples"

We have updated the wording of this sentence to include ‘molecules’ (line 198).
